# Effectiveness of a Multi-Component Smoking Cessation Support Programme (McSCSP) for Patients with Severe Mental Disorders: Study Design

**DOI:** 10.3390/ijerph110100373

**Published:** 2013-12-24

**Authors:** Maria Paz Garcia-Portilla, Leticia Garcia-Alvarez, Pilar Alejandra Saiz, Eva Diaz-Mesa, Gonzalo Galvan, Fernando Sarramea, Josefa Garcia-Blanco, Edorta Elizagarate, Julio Bobes

**Affiliations:** 1 Departmento de Psiquiatría, Universidad de Oviedo, 33006 Oviedo, Spain; E-Mails: albert@uniovi.es (M.P.G.-P.); bobes@uniovi.es (J.B.); 2 Centro de Investigación Biomédica en Red de Salud Mental, CIBERSAM, Universidad de Oviedo, 33006 Oviedo, Spain; E-Mails: lettti@gmail.com (L.G.-A.); evamdmesa@gmail.com (E.D.-M.); 3 Universidad Nacional de la Patagonia Austral, Puerto San Julián, 9310 Santa Cruz, Argentina; E-Mail: galvan.patrignani@gmail.com; 4 Instituto Interuniversitario de Postgrado en Salud, Santa Rosa, 6300 La Pampa, Argentina; 5 Complejo Hospitalario de Jaén, Equipo de Salud Mental de Andújar, 23740 Jaén, Spain; E-Mails: fernandosarramea@hotmail.com (F.S.); p.gblanco@hotmail.com (J.G.-B.); 6 Unidad de Psicosis Refractarias, Hospital Psiquiátrico de Álava, 01006 Vitoria, Spain; E-Mail: edorta.elizagaratezabala@osakidetza.net; 7 Centro de Investigación Biomédica en Red de Salud Mental, CIBERSAM, Universidad del País Vasco, 48940 Leioa-Bizkaia, Spain

**Keywords:** schizophrenia, bipolar disorder, transdermal nicotine patches, varenicline, bupropion

## Abstract

Only a few studies have examined the efficacy and safety of smoking cessation programmes in patients with mental disorders. The aim of this paper is to describe in detail the methodology used in the study as well as the Multi-component Smoking Cessation Support Programme in terms of pharmacological treatments and psychological interventions. An open-label 9-month follow-up study was conducted in Spain. A total of 82 clinically stable outpatients with schizophrenia, schizoaffective or bipolar disorder were enrolled. Treatment consisted of a programme specifically developed by the research team for individuals with severe mental disorders. The programme consisted of two phases: (1) weekly individual motivational therapy for 4–12 weeks, and (2) a 12-week active treatment phase. During this phase, at each study visit patients received a one- or two-week supply of medication (transdermal nicotine patches, varenicline or bupropion) with instructions on how to take it, in addition to group psychotherapy for smoking cessation. Evaluations were performed: (1) at the time of enrolment in the study, (2) during the 12-week active treatment phase of the study (weekly for the first 4 weeks and then biweekly), and (3) after the end of this phase (two follow-up assessments at weeks 12 and 24). Evaluations included: (1) smoking history, (2) substance use, (3) psychopathology, (4) adverse events, and (5) laboratory tests. The importance of this study lies in addressing a topical issue often ignored by psychiatrists: the unacceptably high rates of tobacco use in patients with severe mental disorders.

## 1. Introduction

Although smoking rates are declining in the general population in developed countries [1], patients with long-standing mental health disorders are almost twice as likely to smoke as people without such problems [2]. In fact smoking prevalence rates among people with mental disorders (schizophrenia, mood and anxiety disorders) are two to four times higher than in the general population [3,4]. Prevalence is greatest in patients with schizophrenia with rates around 64% followed by patients with bipolar disorder, with rates around 44% [5]. In Spain the reported tobacco use rate in patients with schizophrenia was 54.4% [6] and 51.5% in patients with bipolar disorder [7,8]. These rates are practically twice that of the general Spanish population (26.4%) [9]. 

This exceptionally high prevalence, explained by several biological and psychosocial factors, contributes to the high rates of medical morbidity and mortality in patients with severe mental disorders [6,7,10,11]. Life expectancy was found to be 25 years lower in patients with mental illnesses, mainly due to tobacco-related diseases [12]. In this respect the Spanish Consensus on Physical Health of Patients with Schizophrenia [13] and with Bipolar Disorder [14] rank respiratory disorders (standardized mortality ratio of 3–7 in patients with bipolar disorder) followed by cardiovascular disease as the leading causes of the increased mortality rates in these patients. With respect to morbidity, tobacco use seems to be one of the most important risk factors for the greater risk of coronary disease both in patients with schizophrenia [6,15,16,17] and bipolar disorder [7].

In addition to its negative impact on physical health, tobacco use has been associated with more excitement and agitation symptoms [18,19], greater severity of global psychopathology as measured by the Clinical General Impression (CGI) scale [6], and positive psychotic symptoms [6,20] in patients with schizophrenia, although the effect size was small [6]. In patients with bipolar disorders the results are controversial; while some studies showed tobacco use to be associated with greater disorder severity [21,22] others did not [7]. Recently a relationship was also reported between smoking and use of a greater number of psychopharmaceuticals for the treatment of bipolar disorder [7]. Furthermore, an association was found between tobacco use and greater attempted suicide rates in patients with bipolar disorders [23,24] and with schizophrenia [25]. Some studies have also reported beneficial effects of tobacco on patients’ mental health. Thus improvements in negative symptoms [20,26], spatial working memory and attention deficit [27], and sensory gating [28] have been reported. However we believe that these beneficial effects do not justify maintaining the smoking habit in this population, as tobacco is associated with more than 4,000 toxins and 60 carcinogens, and nicotine can be delivered more safely through approved drugs. 

Studies on the smoking patterns of individuals with severe mental disorders have shown that they started smoking an average of 5 years prior to illness onset [29,30,31], smoked a large number of cigarettes per day [29], had higher plasma nicotine levels than smokers without mental disorders [32,33] and were more nicotine-dependent than general population [16,34,35,36].

While smokers with severe mental disorders have quite low smoking cessation rates, they have demonstrated high levels of motivation to quit [37,38], persistent attempts [39] and good tolerance of short-term nicotine abstinence without significant clinical exacerbation [40,41,42,43]. Despite all the above, only a few studies have examined the efficacy and safety of smoking cessation programmes in patients with mental disorders. For this reason we decided to develop a Multi-component Smoking Cessation Support Programme (McSCSP) tailored to the specific characteristics of individuals with severe mental disorders, as recommended by the European Association of Psychiatry (EPA) in its Position Statement on Smoking and Strategies for Smoking Cessation in People with Mental Illness [44], and to determine its clinical effectiveness. The McSCSP consisted of two phases: (1) weekly individual motivational therapy for 4–12 weeks, and (2) a 12-week active treatment phase. During this phase, at each study visit patients received a one- or two-week supply of medication with instructions on how to take it, in addition to group psychotherapy for smoking cessation.

The aim of this paper is to describe in detail the methodology used in the study, *i.e.*, inclusion and exclusion criteria, clinical evaluations including smoking history, substance use, psychopathology, adverse events, anthropometrics, vital signs, and laboratory tests, as well as the McSCSP in terms of pharmacological treatments and psychological interventions.

## 2. Methods

### 2.1. Study Design

This is a non-randomized, open-label (both the researchers and participants know which treatment is being administered), 9-month follow-up study conducted at three sites in Spain (Oviedo, Vitoria, and Jaén) between March 2011 and June 2013. The study was conducted at two Mental Health Centres (Oviedo and Jaén) and at the Refractory Psychosis Unit where outpatients from the city of Vitoria were referred for the study.

The Clinical Research Ethics Committee of Hospital Universitario Central de Asturias in Oviedo, Spain approved the study protocol. Written informed consent was obtained from all subjects prior to enrolment.

### 2.2. Subjects

The sample size needed for this study was determined using the EpiInfo v.6.0 program. Considering a population of 150 patients with severe mental disorder for each of the three psychiatrists participating in the study, a 6-month successful smoking cessation rate of 12% of the patients at six months, and a confidence interval level of 95%, a sample size of *n* = 23 per centre is needed to achieve the stated goals.

**Figure 1:**
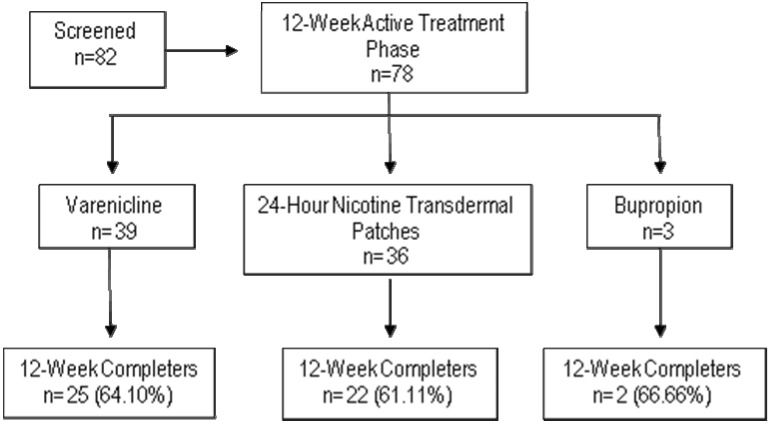
Patients’ disposition.

A total of 82 outpatients with schizophrenia, schizoaffective or bipolar disorder on maintenance treatment were enrolled in the study (74.4% schizophrenia or schizoaffective disorder and 25.6 bipolar disorder). Four of them dropped-out before initiating the active treatment phase. Of the 78 patients who were included in the active phase of treatment, 50% received varenicline, 46.2% 24-hour nicotine transdermal patches, and 3.8% bupropion SR. A total of 62.8% of the patients completed the 12-week active treatment phase. There was no statistically significant differences in the retention rate among the three drugs (see Figure 1).

Inclusion criteria were: (1) patients with a confirmed DSM-IV diagnosis of schizophrenia, schizoaffective or bipolar disorder, clinically stable (i.e., without hospitalization or acute exacerbation) in the six months prior to enrolment in the study and on maintenance treatment; (2) currently smoking 15 or more cigarettes/day without a period of smoking abstinence longer than one month during the previous year; (3) Fagerström Test for Nicotine Dependence score ≥4 (moderate); (4) breath carbon monoxide (CO) level >9 particles per million (ppm); (5) between 18 and 65 years of age; (6) without suicidal ideation; and (7) written informed consent to participate in the study.

Patients were excluded if they had (1) a total score >70 on the Positive and Negative Symptoms Scale (PANSS) for patients with schizophrenia, or >14 on the Hamilton Depression Rating Scale (HDRS) or >6 on the Young Mania Rating Scale (YMRS) for patients with bipolar disorder; (2) serious suicidal behaviour or thoughts in the last six months; (3) severe and unstable somatic illness including but not limited to acute myocardial infarction, unstable arrhythmia, stroke; (4) history of organic brain damage including epilepsy, tumours, head injuries with significant cognitive impairment; (5) significant renal impairment (creatinine ≥ 1.5 mg/dL); and (6) liver function tests more than twice the upper limit of normal values.

### 2.3. Diagnostic Interview

Diagnosis was confirmed by the psychiatrists participating in the study using the Schizophrenia, Schizoaffective and Bipolar sections of the Spanish version of the Structured Clinical Interview for DSM-IV Axis I Disorders (SCID-I) [45].

### 2.4. Demographic and Clinical Data

Demographic and clinical data were collected at baseline. Demographic data included: birth date, age, gender, civil status, educational level, main occupation, and current working status. Clinical data included: primary diagnosis, secondary diagnosis, length of illness, first episode, previous suicide attempts, and current psychopharmacological treatment. Changes in psychopharmacological treatment were recorded at every visit. 

### 2.5. Clinical Evaluations

Psychometric and biological evaluations included smoking history, substance use, psychopathology, adverse events, anthropometrics, vital signs and laboratory tests (see Table 1). For all instruments used in the study we employed the validated Spanish versions with the exception of the Columbia Suicide Severity Rating Scale (CSSRS), which is currently being validated (Project PI: Pilar A. Saiz), and the Richmond test which, although it has not been validated in Spanish, is widely used in our country [46].

**Table 1:** Psychometric and biological evaluations.

Area of Assessment		Psychometric Instruments/ Biological Parameters	Inclusion Criteria	Exclusion Criteria
Smoking history	Pattern of tobacco use	Age at first use Number of years smoked Smoking pack-year (SPY) Cigarette brand Cigarettes per day (CPD) Breath carbon monoxide (CO) level	≥15 cigarettes/day >9 ppm	
	Nicotine dependence	Fagerström Test for Nicotine Dependence (FTND) Glover-Nilsson Smoking Behavioral Questionnaire	Total score ≥ 4	
	Motivation to quit	Richmond test University of Rhode Island Change Assessment (URICA) scale		
Substance use	Caffeine	Daily consumption		
	Other	Drug Use Table—Individual Substances of the Addiction Severity Index 6th version (ASI6)		
Psychopathology	Schizophrenia or schizoaffective patients	Positive and Negative Syndrome Scale (PANSS)		Total score > 70
	Patients with bipolar disorder	Hamilton Depression Rating Scale (HDRS) Young Mania Rating Scale (YMRS)		Total score > 14 Total score > 6
	Both types of patients	SCID-I: Schizophrenia, Schizoaffective and Bipolar sections Columbia Suicide Severity Rating Scale (CSSRS) Clinical Global Impression (CGI)	Meeting criteria for one disorder	Serious suicidal behaviours or thoughts
Adverse events		UKU Side Effects Rating Scale (UKU)		
Biological evaluation	Anthropometrics	Weight, height, body mass index (BMI), waist circumference		
	Vital signs	Blood pressure, heart rate		
	Laboratory tests	Renal function tests: creatinine, BUN, glomerular filtration rate Liver function tests: aspartate aminotransferase (AST), alanine aminotransferase (ALT), gamma glutamyl transpeptidase (GGT), total bilirubin (TBIL) Lipid profile: triglycerides, total cholesterol, HDL cholesterol, LDL cholesterol		Creatinine ≥ 1.5 Liver function tests > 2 × the upper limit of normal

#### 2.5.1. Smoking History

Pattern of tobacco use was described with the following parameters: age at first use, number of years smoked, pack-years, cigarette brand, cigarettes per day (CPD), and breath carbon monoxide (CO) level. 

Smoking packyears (SPY): Tobacco exposure has a cumulative effect so it is necessary to consider not only current consumption but also global lifetime consumption. SPY is a numerical value that represents the amount an individual has smoked over his/her life. The following formula is used to calculate it: (number of cigarettes per day_A_ × years of consumption of amount_A_/20) + (number of cigarettes per day_B_ × years of consumption of amount_B_/20) + ... + (number of cigarettes per day_n_ × years of consumption of amount_n_/20). A calculator can be downloaded from http://smokingpackyears.com/calculate [47].

Cigarettes per day (CPD): The number of CPD can be considered a face valid measure of nicotine dependence with low predictive abstinence value [48]. As there is no consensus on how to best define “light” and “heavy” smokers, we classified subjects into three categories according to the following classification: self-reported CPD <10 = light smoker; 11–20 = moderate smoker; and >20 = heavy smoker.

Breath CO levels: Breath CO was measured with a portable piCO^simpleTM^ Smokerlyzer^®^ monitor (Bedfont Scientific Ltd., Kent, UK). In keeping with the majority of studies we used a cutoff point for current smokers of 9 ppm. Since smokers have diurnal variation in CO [49,50] it was measured between 9 a.m. and 11 a.m.

Nicotine dependence: This was evaluated using the Fagerström Test for Nicotine Dependence (FTND) [50] and the Glover-Nilsson Smoking Behavioral Questionnaire [51]. The FTDN comprises six items that evaluate the degree of nicotine dependence. The FTDN total score ranges from 0 to 10 and categorizes patients as having mild (0–3), moderate (4–7), and severe (8–10) nicotine dependence. It was recently recommended by the European Psychiatric Association for use in daily clinical practice [44].

The Glover-Nilsson test consists of 11 items that capture behavioural nicotine dependence. It classifies behavioural dependence into four levels according to total score: 0–11 = mild; 12–22 = moderate; 23–33 = strong; 34–44 = very strong.

Motivation to quit: The Richmond test [52] and the University of Rhode Island Change Assessment (URICA) scale [53] were used to measure the individual’s motivation to quit smoking and his/her readiness to change, respectively. The Richmond test is a 4-item instrument that evaluates motivation to quit smoking. Total score ranges between 0 and 10. Scores between 0 and 4 indicate low motivation; 5–6 moderate motivation; and 7–10 high motivation to quit.

The URICA is a 32-item scale that contains four 8-item subscales measuring the stages of change: Precontemplation (PC), Contemplation (C), Action (A), and Maintenance (M). A continuous “Readiness to Change” score can be obtained using the following formula [(Avg C + Avg A + Avg M) − Avg PC], where Avg represents the average score of each of the four subscales in order to evaluate an individual’s level of motivation for change. Since there are no normative scores for the Spanish population we decided to use subscale scores instead of the readiness to change score to determine each patient’s stage of the change.

#### 2.5.2. Substance Use

The use of the following substances was assessed with the Drug Use Table—Individual Substances of the Spanish Addiction Severity Index 6th version (ASI6) [54]: marijuana, sedatives, cocaine, stimulants, hallucinogenics, heroin, methadone, other opiates, and inhalants. For each substance we determined the age at first use, years of regular use, if used 50 or more days (lifetime), use in past 30 days, and if used as treatment (last 30 days). In addition we recorded daily caffeine consumption by asking about daily consumption of coffee, tea, cola and energy drinks (Burn, Red Bull, Fire, *etc.*). Caffeine consumption was recorded as number of cups of coffee per day based on the following equivalence: 1 cup of coffee = 1 energy drink = 2 cups of tea = 3 colas [55].

#### 2.5.3. Psychopathology

In patients with schizophrenia we used the PANSS [56], which measures the severity of positive, negative, and general psychopathology symptoms. In patients with bipolar disorder we used the HDRS [57] and the YMRS [58], which rate the severity of depressive and manic symptoms, respectively. In both types of patients we utilized the CGI [59] for assessing severity and change in global psychopathology, and the CSSRS to evaluate suicidal thoughts and behaviours.

#### 2.5.4. Adverse Events

Unwanted side effects of pharmacological treatment were assessed by means of the UKU Side Effects Rating Scale (UKU) [60]. In addition, we will also get information about them through the psychometric ratings, anthropometric measures, laboratory results and spontaneous patient’ self-reports.

#### 2.5.5. Anthropometrics, Vital Signs, and Laboratory Tests

Weight and height were measured without jackets and shoes. Body mass index (BMI) was calculated as body weight (kg) divided by the square of height (m^2^). Waist circumference was measured at the high point of the iliac crest and at the level of the umbilicus. Heart rate was measured as number of beats per minute using the wrist palpation method. Blood pressure consisted of a single seated determination. Blood samples were collected if patients confirmed having fasted for at least 8 h. Creatinine, BUN, glomerular filtration rate, aspartate aminotransferase (AST), alanine aminotransferase (ALT), gamma glutamyl transpeptidase (GGT), total bilirubin (TBIL), triglycerides, total cholesterol, and HDL and LDL cholesterol were tested.

### 2.6. Assessments

All subjects were evaluated at the time of enrolment in the study before starting the motivational therapy. During the 12-week active treatment phase patients were assessed weekly for the first 4 weeks and then biweekly. After the end of this phase, two follow-up assessments were done at weeks 12 and 24 (study weeks 24 and 36 respectively). Table 2 displays the timing and the parameters evaluated.

**Table 2:** Task schedule.

Study Phase	Motivation	Active Treatment									Follow-up	
***Visit number***	MV1	V0	V1	V2	V3	V4	V5	V6	V7	V8	FU- V1	FU- V2
***Week***	w-12 to w-4	w0	w1	w2	w3	w4	w6	w8	w10	w12	w24	w36
Inclusion/Exclusion criteria	X											
Informed consent	X											
Demographic data	X											
Clinical data	X											
SCID-I: Schizophrenia, Schizoaffective and Bipolar sections	X											
***Smoking history***												
Pattern of tobacco use	X											
Cigarettes per day (CPD)	X		X	X	X	X	X	X	X	X	X	X
Breath CO level	X	X	X	X	X	X	X	X	X	X	X	X
Fagerström Test for Nicotine Dependence (FTND)	X	X	X	X	X	X	X	X	X	X	X	X
	X	X	X	X	X	X	X	X	X	X	X	X
Glover-Nilsson Smoking Behavioral Questionnaire	X	X										
	X	X										
Richmond test												
University of Rhode Island Change Assessment (URICA) scale												
***Substance use***												
Daily caffeine consumption	X	X	X	X	X	X	X	X	X	X	X	X
Drug Use, Table from the Addiction Severity Index 6th version (ASI6)	X	X	X	X	X	X	X	X	X	X	X	X
***Psychopathology***												
Positive and Negative Syndrome Scale (PANSS)	X	X	X	X	X	X	X	X	X	X	X	X
	X	X	X	X	X	X	X	X	X	X	X	X
Hamilton Depression Rating Scale (HDRS)	X	X	X	X	X	X	X	X	X	X	X	X
	X	X	X	X	X	X	X	X	X	X	X	X
Young Mania Rating Scale (YMRS)	X	X	X	X	X	X	X	X	X	X	X	X
Columbia Suicide Severity Rating Scale (CSSRS)	X	X	X	X	X	X	X	X	X	X	X	X
			X	X	X	X	X	X	X	X	X	X
Clinical Global Impression (CGI)												
Severity												
Change												
***Adverse events***												
UKU Side Effects Rating Scale (UKU)	X	X	X	X	X	X	X	X	X	X	X	X
											
***Biological evaluation***												
Anthropometrics and vital signs	X	X	X	X	X	X	X	X	X	X	X	X
Laboratory tests	X					X				X	X	X

Notes: FU: follow-up; M: motivation; V: visit; w: week; w-12 to w-4: between 12 and 4 weeks before starting Active Treatment (V0, w0).

### 2.7. Statistical Plan

We will determine short-term (at the end of the 12-week active treatment phase), and 3- and 6-months after active treatment outcomes. The primary outcome measure will be smoking cessation, a composite measure formed by the patient's self-report of previous 7-day abstinence confirmed by breath CO levels ≤ 9 ppm. It will be also considered as main outcome measure the proportion of subjects with at least 50% reduction in the number of cigarettes per day. Secondary outcome measures will be safety, including changes in the symptoms of the primary illness, and tolerability.

### 2.8. Treatment

#### 2.8.1. Pharmacological Treatment

The pharmacological treatments used in the study were those approved for smoking cessation (bupropion sustained release, nicotine replacement therapy (transdermal patches) and varenicline) and considered first-line options by the US Public Health Service in 2008 [61]. Recently the European Psychiatric Association (EPA) [44] recommended drug treatment with nicotine, varenicline or bupropion for even a mild degree of tobacco dependence in every psychiatric patient. However, it should be pointed out that, in its Therapeutic Prescribing Guidelines, the Agencia Española de Medicamentos y Productos Sanitarios does not include varenicline among the drugs approved for smoking cessation and specifically contraindicates the use of bupropion in patients with bipolar disorder [62]. 

For each patient the drug was chosen based on the clinical characteristics of his/her mental disorder, smoking pattern and previous smoking cessation experiences, somatic comorbidities and their pharmacologic treatments, as well as patient preference. Varenicline was given according to the usual schedule, *i.e.*, 0.5 mg/day for the first 3 days, 0.5 mg BID on days 4–7, and 1 mg BID for the remaining 11 weeks. Bupropion SR was given as recommended, *i.e.*, 150 mg/day for the first 6 days and 150 mg BID for the remaining treatment period. Twenty-four-hour nicotine transdermal patches were given to patients at doses of 14, 21, 28 or 35 mg based on their tobacco use during the last 12 weeks.

In the case of psychopathological exacerbation or serious adverse events the pharmacological treatment was discontinued and the subject was withdrawn from the study. 

#### 2.8.2. Psychological Interventions

The study included two types of sequential psychological interventions. Firstly those patients willing to quit smoking and participate in the study received individual motivational therapy to prepare them for the active treatment phase. Secondly group psychotherapy was provided as part of the active treatment phase along with pharmacotherapy.

Motivational therapy: Those smokers who wanted to quit received individual motivational therapy before entering the active treatment phase. The programme was flexible, lasting between 4 and 12 sessions depending on each subject’s characteristics, and was based on the motivational interviewing technique [63]. Its main objective was to help patients move forward through the Stages of Change Model [64] to the stages of Contemplation or Action. The main issues addressed during the sessions were (1) pros and cons of smoking, (2) health and financial burden of smoking, and (3) concerns about quitting. 

We included the financial burden of smoking because we believe that in patients with severe mental disorder this is a very important point as shown by Lawn [65]. Many of them are financially dependent on a mental disability benefit and spend a large part of their monthly income on cigarettes.

Group therapy: A specific intensive 12-week manualized group therapy programme for individuals with severe mental was developed by the research team based on available smoking cessation guidelines and our own experience. The programme is suitable for delivery by healthcare providers experienced in working with patients with severe mental disorders and in conducting psychoeducational groups, including psychiatric nurses, psychologists and psychiatrists. Session content covered decisional balance, nicotine withdrawal and psychiatric symptoms, weight gain, control techniques, advantages and disadvantages of the pharmacological treatment options, improving adherence, and preventing relapses. In addition to group therapy, patients had to do homework that was reviewed at the beginning of the next session. Each treatment session lasted 60–75 min and included six to eight patients who achieved the Contemplation or Action stages of the Prochaska and DiClemente Model [64]. 

## 3. Discussion

This study tries to determine the effectiveness of a McSCSP specifically developed for use in individuals with severe mental disorders. The importance of this study lies in addressing a topical issue often ignored by psychiatrists: the unacceptably high rates of tobacco use in patients with severe mental disorders. The EPA—Position Statement on Smoking and Strategies for Smoking Cessation in People with Mental Illness concludes that “since tobacco dependence is a dependence disorder, psychiatrists are the experts in performing interventions in this area. It is their duty to do so in view of the major impact of tobacco dependence on, for example, the metabolism of psychotropic treatments, morbidity (such as lung cancer) and mortality” [44]. Furthermore, the relative absence of published results in this field adds more value to our study. In this study we have shown that such programmes are feasible and can be implemented in centers treating patients with severe mental illness since 66% of them completed the active treatment phase.

An advantageous feature of the study is its external validity and therefore the generalizability of our results. The inclusion and exclusion criteria used allowed us to enrol “real world” patients seen in daily clinical practice. Only those patients with an acute exacerbation of their mental illness or in whom the three study drugs were contraindicated were excluded from the study. Although we utilized the Spanish version of the URICA test [53] to identify each patient's stage of change according to the Prochaska and DiClemente Model [64], this information was not considered an inclusion or exclusion criterion due to the lack of Spanish normative scores or cutoff scores, data in people with severe mental disorders, and published doubts about its usefulness [66,67]. We hope to identify whether any of the stages of change is associated with higher rates of smoking cessation.

Another strength of this study is the sample size. Although 82 patients is not a very large sample, to our knowledge the majority of the published studies in patients with severe mental disorders included far fewer patients or were case reports. However, the lack of a control group may be considered an important shortcoming. The open-label design of the study could be also considered a limitation of the study. However, this study was intended to be a pragmatic trial of the effectiveness of a multi-component programme specifically designed for individuals with severe mental disorders rather than a clinical trial designed to determine the comparative efficacy of the approved drugs for smoking cessation. 

One strong point of our study in comparison with other smoking cessation studies is the comprehensive clinical evaluation that was done. In addition to taking an exhaustive smoking history we assessed the use of other substances, the psychopathology severity including suicidal thoughts and behaviours, and adverse events using validated psychometric instruments. Standard laboratory tests were also performed. Conversely, a weak point is the lack of collection on somatic comorbidities. However, since we have anthropometric data and laboratory results we can obtain information on obesity, metabolic syndrome and cardiovascular risk, the most frequent somatic comorbidities in these patients and highly associated with their premature mortality.

Finally, the McSCSP was developed with the aim of providing mental healthcare professionals with an intervention that could be applied in specialized routine clinical practice. It may be said that 60 to 75 min is too long for implementation in standard mental health centres. However since it is designed for use in severely ill patients in specialized medical facilities (psychiatric units), and given the potential benefits to both the healthcare system and patients, as well as the group modality of the intervention (60–75 min for 6 to 8 patients), this should not constitute a barrier, or worse, an excuse not to address the problem. This will be of interest if we are able to demonstrate that our smoking cessation programme is safe and effective in patients with severe mental disorders and should thus be disseminated for use in everyday clinical practice in Spain.

## 4. Conclusions

The prevalence rate of tobacco dependence among patients with severe mental disorders is about twice that of people without these disorders. In addition, tobacco is one of the most important avoidable factors contributing to the high rates of morbidity and mortality seen in these patients. Therefore, psychiatrists, clinical psychologists and psychiatric nurses, as experts in addiction treatment, should motivate and help their patients to quit smoking
